# Comparative Characterisation of Proliferation and Apoptosis of Colonic Epithelium after Electron Irradiation with 2 GY and 25 GY

**DOI:** 10.3390/ijms25021196

**Published:** 2024-01-18

**Authors:** Grigory Demyashkin, Elza Karakaeva, Susanna Saakian, Natalia Tarusova, Amina Guseinova, Anita Vays, Konstantin Gotovtsev, Dmitrii Atiakshin, Petr Shegai, Andrey Kaprin

**Affiliations:** 1 Laboratory of Histology and Immunohistochemistry, I.M. Sechenov First Moscow State Medical University (Sechenov University), 119991 Moscow, Russia; 2 Department of Pathomorphology, National Medical Research Centre of Radiology, Ministry of Health of Russia, 125284 Moscow, Russia; 3 Research and Educational Resource Center for Immunophenotyping, Digital Spatial Profiling and Ultrastructural Analysis Innovative Technologies, RUDN University, 117198 Moscow, Russia

**Keywords:** radiotherapy, electrons, irradiation, colon, caspase-3, Ki-67

## Abstract

Development of new techniques for multimodal treatment and diagnostics of various neoplasms and the improvement of current techniques can significantly increase the life expectancy of patients with carcinomas of the colon and abdominal-cavity organs, since prevention of various side effects of radiation therapy is one of the main problems of oncological care. Electron irradiation is one of the most promising types of radiation therapy. There are no data on proliferation and apoptosis of the colon epithelium after irradiation with electrons, especially in different modes (single and summary). Morphological evaluation of apoptosis and proliferation of colonic epithelium after local irradiation with electrons were conducted at doses of 2 Gy (Gray) and 25 Gy. Colon fragments from sexually mature Wistar rats (*n* = 50, body weight 200 ± 10 g) were divided into three groups: I—control (*n* = 10); II—experimental group (*n* = 20; local single electron irradiation at a dose of 2 Gy); III—experimental group (*n* = 30) with local fractional irradiation with electrons at a total dose of 25 Gy. They were studied using light microscopy using hematoxylin and eosin staining and immunohistochemical reactions with antibodies to Ki-67 and caspase-3 (Cas3). Morphological disorders were accompanied by increased expression of pro-apoptotic molecules (caspase-3), and the period of regeneration by proliferative marker (Ki-67). Colon electron irradiation led to disturbances in the histoarchitecture of varying severity, and an increase in cell apoptosis was observed (increased expression of caspase-3 and decrease in Ki-67). In addition, modulation of the PI3K/AKT and MAPK/ERK signalling pathways was detected. The most pronounced destructive changes were observed in the group of 25 Gy fractionated electron irradiation.

## 1. Introduction

The improvement and development of multimodal treatment and diagnostic methods for various cancers is significantly prolonging the life of patients with colonic cancer; therefore, prevention of the side effects of anti-tumour therapy is one of the main problems in oncological care [1,2]. Most patients are treated with radiotherapy in addition to chemotherapy. Extracorporeal pelvic radiotherapy (ETLT) is more commonly used for neoplasms of the gastrointestinal, urinary, and reproductive systems. ETLT is used as a neoadjuvant therapy before surgery, together with chemotherapy, or as a stand-alone treatment [3]. Radiation has both a direct effect on cells, damaging DNA and causing cell death, and an indirect effect through ionisation of intracellular molecules [4]. The cytotoxic effect is manifested not only in atypical cells but also in the paratumoral region and in the surrounding normal tissue. If a tumour of one or more pelvic organs is irradiated, post-irradiation damage may occur in any of these organs due to their anatomical proximity.

The intestine is one of the most radiosensitive organs [5]. Firstly, the intestinal epithelium is one of the most frequently renewed and rapidly dividing tissues. Secondly, due to its long length, there is a risk of extensive damage. Stem cells are the most sensitive to ionizing radiation due to their high mitotic activity [6,7].

Radiation-induced intestinal lesions are common complications in oncology, for example, such as post-radiation colitis [8]. It was also interesting to study the activity of the genes of the molecular pathways PI3K/AKT and MAPK/ERK, which most often respond to irradiation [6].

One of the most promising forms of radiotherapy is electron-beam irradiation, which, according to a number of researchers, has a less damaging effect on paratumoral tissues, thus preserving the structural and functional status of the colon in particular, and also providing prevention of post-radiation damage. Therefore, to assess the functional status of the colon, it is necessary to study the molecular mechanisms of epitheliocyte proliferation and apoptosis.

The aim of the study is as follows: To provide a morphological evaluation of apoptosis and proliferation of colonic epithelium after local irradiation with electrons at doses 2 Gy and 25 Gy.

## 2. Results

Macroscopic examination of the colon of the experimental group after local single electron irradiation with 2 Gy showed no signs of an inflammatory process, tumour growth, or destructive changes. Histological examination after local single electron irradiation with 2 Gy of the colon slides on the fifth day showed a slight reduction in goblet cells with a tendency towards recovery.

Macroscopic examination of the colon of the experimental group on the fifth day after local fractional-electron irradiation with 25 Gy showed hyperemia of the mucosa without pronounced destructive change.

**Figure 1:**
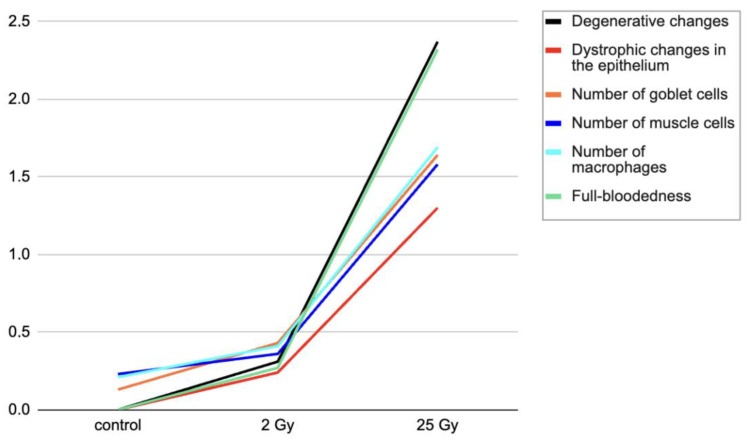
Morphological characteristics of the colon after local electron irradiation at doses 2 Gy and 25 Gy, in points.

Microscopic examination on the fifth day after local electron irradiation at local fractional electron irradiation at a summary dose of 25 Gy revealed moderate degenerative changes of the colonic mucosa, and hypertrophy and dystrophic changes of some epitheliocytes with reduction of mitotic figures in comparison with the control group. A reduction of goblet cells (63.17 ± 1.87 vs. 21.64 ± 1.37) was observed over a large area. The wall thickness and diameter of the colon were also reduced. The thickness of the muscular lamina of the mucosa and muscularis was slightly reduced due to reduction and dystrophic changes of some smooth muscle cells. A moderate cellular inflammatory infiltration, mainly of lymphocytes and macrophages, was found in the stromal component. The vascularisation index was increased, with full blood flow and congestion in some blood vessels (Table 1 and Figure 1).

It should be emphasised that the disruption of the colonic histoarchitecture caused by electron irradiation, accompanied by damage to intestinal crypts with a high regenerative potential, made it difficult to count cell populations.

According to the quantitative analysis carried out, the colon has a moderate radioresistance, which is partly in agreement with studies by other authors using other irradiation methods [4].

**Figure 2:**
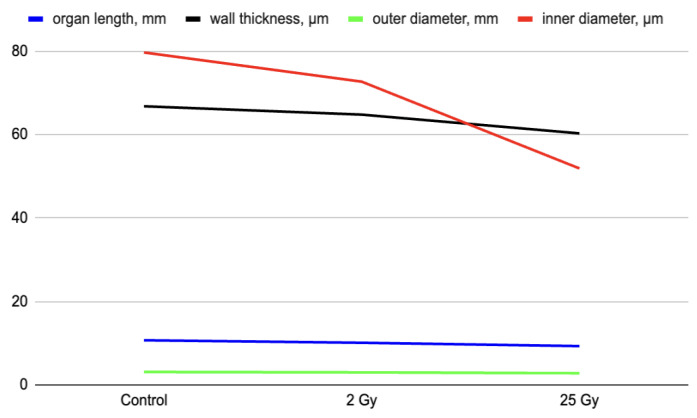
Morphometric characteristics of the colon after local electron irradiation at doses 2 Gy and 25 Gy, in points.

The morphometric study of the colon after single local electron irradiation at dose 2 Gy showed highly insignificant histomorphometric changes in some parameters (Table 1 and Figure 2).

The morphometric study of the colon after fractional irradiation with electrons at local fractional electron irradiation at the summary dose 25 Gy showed insignificant histomorphometric changes in some parameters (Table 2). For example, when comparing the thickness of the colon wall and the internal diameter, which reflects the width of the organ lumen, we found an insignificant decrease in the parameters. The intraluminal diameter of the colon after electron irradiation decreased by 13–20% compared to the control group (*p* < 0.05). At the same time, measurements of colon length and outer diameter showed no statistically significant difference (*p* > 0.05).

The number of caspase-3- and Ki-67-positive cells was examined using immunohistochemistry.

**Figure 3:**
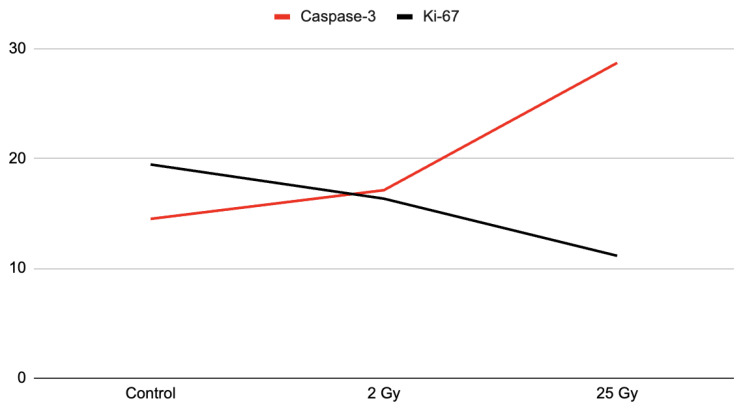
Number of IHC-positive colon epitheliocytes after local electron irradiation at doses 2 Gy and 25 Gy, in points.

On the fifth day, after a single electron irradiation at a dose of 2 Gy, the number of caspase-3- and Ki-67-positive cells almost approached the control values. No statistically significant difference was observed, *p* > 0.05 (Table 3 and Figure 3).

After irradiation with 25 Gy, the number of caspase-3-positive epithelial cells increased 1.97-fold compared to the control values (Table 3, Figure 3). At the same time, normal histoarchitecture of the colon crypts and a few caspase-3-positive cells were observed in the colon samples of the control group. The proliferation index (Ki-67) in the intestinal crypts of the colon, after local irradiation with electrons at a local fractional electron irradiation at summary dose 25 Gy, was reduced by 1.74 times compared to the control group (Table 3 and Figure 3).

**Figure 4:**
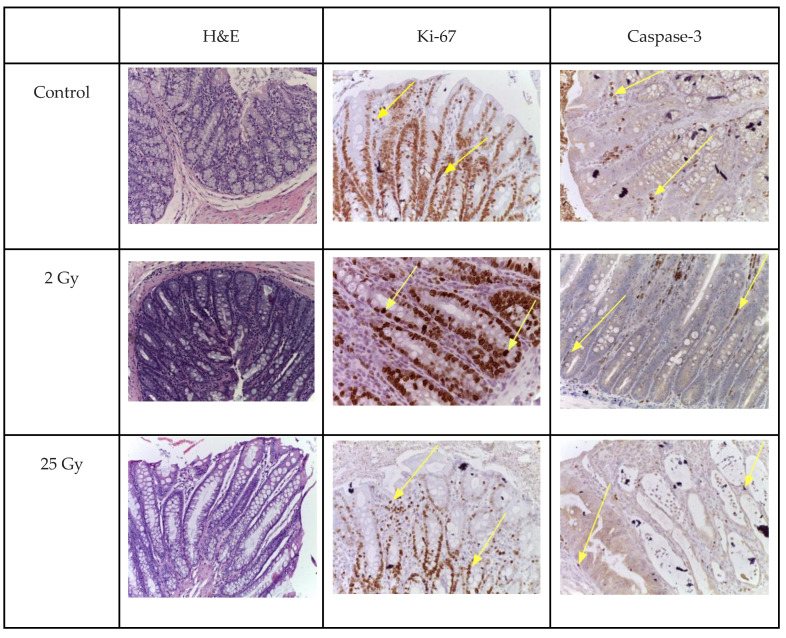
Colon after local electron irradiation at doses 2 Gy and 25 Gy. Immunohistochemical reactions with antibodies to Ki-67 (arrow) and caspase-3 (arrow); staining hematoxylin; ×200.

The data obtained indicate that the damage to the intestinal crypts of the colon induced by irradiation with electrons at local fractional electron irradiation is accompanied by a decrease in the number of progenitor cells, and, as a consequence, leads to a decrease in the “survival rate” of the crypts (Figure 4).

**Figure 5:**
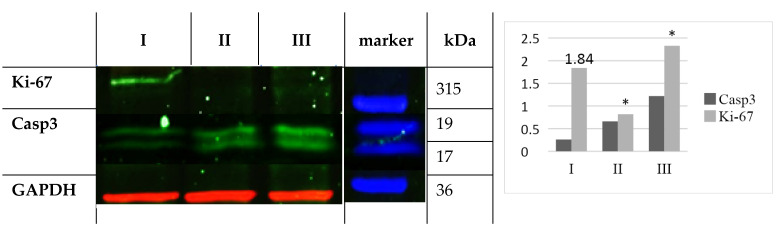
Western blot analysis of Ki-67 and Caspase 3 in the colon of the control and experimental groups. Ki-67 and Caspase 3 expression was measured in nmol pNA/mg protein C: control (I); 2 Gy (II); 25 Gy (III). Glyceraldehyde-3-phosphate dehydrogenase (GAPDH) immunostaining was used as a standard. (**Left**)—visualization (immunochemiluminescence); (**Right**)—densitometric analysis of Western blot. *—statistically significant comparison with control.

When determining the protein content of Ki-67 (315 kDa) and caspase-3 (19 kDa) using Western blot, statistically significant differences were found between the control and group III at the end of the experiment (Figure 5).

**Figure 6:**
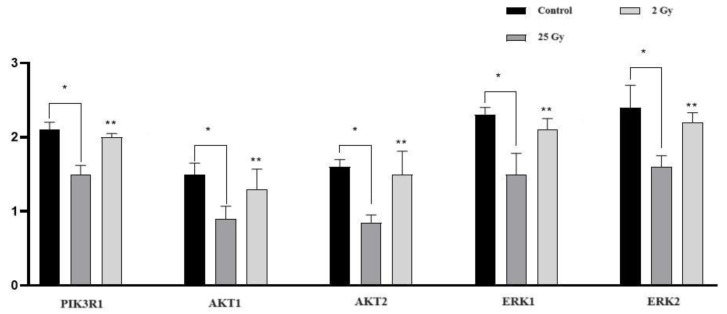
Expression of genes of the PI3K/AKT and MAPK/ERK signalling pathways in the colon of rats five days after local electron irradiation at a summary dose of 25 Gy and a dose of 2 Gy. The abscissa axis shows the studied genes in the control and experimental groups; the ordinate axis shows the relative gene expression. Data are presented as means and confidence intervals. Statistically significant differences between groups: * control vs. 25 Gy, ** 2 Gy vs. 25 Gy; *p* < 0.01.

Using RT-PCR, we studied the activity of genes in the PI3K/AKT and MAPK/ERK signalling pathways. In the colon, 5 days after electron irradiation at a summary dose of 25 Gy, a statistically significant decrease in the relative expression of the genes PIK3R1, AKT1, AKT2, ERK1, and ERK2 was found compared to the control group. After 2 Gy electron irradiation, the expression of the above genes did not differ from the control group, but was statistically significantly higher than in group III (Figure 6).

## 3. Discussion

The present work is devoted to the study of the effect of fractional local irradiation with electrons at a local fractional electron irradiation, at a single dose of 2 Gy and summary dose 25 Gy, on the colonic epithelium of experimental animals.

The present work is devoted to the study of morphofunctional changes in the crypt and the features of proliferation and apoptosis of the colon epithelium after electron irradiation at a dose of 2 Gy. Comparative analyses were performed at two time points, taking into account cycles of colon epithelium proliferation [9,10].

Against the background of local fractional irradiation with local fractional electron irradiation in the colon there are degenerative changes of the mucous membrane, decreases in the number of epitheliocytes, reduction of goblet cells and smooth myocytes, inflammatory infiltration, and hemodynamic changes leading to a violation of local homeostasis. A morphometric study of transverse sections of the colon showed a slight decrease in organ length, wall thickness, and external and internal diameters. When comparing the depth and extent of the pathomorphological changes detected, they were less pronounced in contrast to other types of irradiation [11,12].

We found that the activity of the PI3K/AKT and MAPK/ERK signalling pathway genes correlated with the radiation dose. This may be due to cell death as a result of exposure to electron irradiation. However, it is possible that these are the pathways that respond to electron irradiation in the colon first. Similar results were obtained by other authors [6].

The development of radiation-induced colitis depends on a number of factors, including those responsible for the regulation of the cell cycle of the colonic epithelium, such as caspases, Ki-67, PCNA (proliferating cell nuclear antigen), p53, PUMA (p53 upregulated modulator of apoptosis), etc., as well as a number of other factors.

To assess the regenerative potential after electron exposure, an immunohistochemical study of actively dividing cells of intestinal crypts was performed by determining the DNA of the M-phase of mitosis using biomarkers Ki-67, as well as the terminal stage of apoptosis—caspase-3. The difference in the radiosensitivity of stem and progenitor cells related to DNA damage pathways and cell-cycle regulation was taken into account.

According to some authors, the majority of active stem cells undergo apoptosis in the first day after 12 Gy of X-ray radiation, and then, as a rule, survive due to the effective ability to regenerate after 48 h [13].

The observed increase in the number of caspase-3-positive epitheliocytes was attributed to the cytotoxic action of electrons. However, these changes were weaker compared to other types of irradiation [14]. It should be emphasised that the expected rapid recovery of the pool of epitheliocytes and progenitor cells did not occur, as the fractionation mode was used.

The increase in the activity of the terminal effector caspase-3 is due to the release of cytochrome from the mitochondria, which leads to the multimerisation of Bax and the initiation of the apoptotic cascade, which is implemented through the intrinsic and extrinsic pathways [9,15].

One of the mechanisms of crypt recovery after exposure to electrons is related to their ablative effect on the epithelium, which is in contrast to other types of irradiation, which allows us to assign epitheliocytes to radioresistant populations. In addition, damage to progenitor cells results in delayed repopulation of intestinal crypts.

Activation of either of these mechanisms can lead to a marked decrease in Ki-67 expression and correlate with apoptosis—a dramatic increase in caspase-3.

The regenerative process after electron irradiation, like the effects in other species, appears to consist of three phases: apoptotic (2–4 days), proliferative (2–4 days), and normalization (4–7 days) [16].

Another equally important aspect of the study was to assess the degree of intoxication caused by fractional irradiation with local fractional electron irradiation at a summary dose of 25 Gy. It was found that, unlike other types of irradiation, exposure to electrons does not lead to pronounced changes in the functional status of the colon (maintenance of water–electrolyte balance, etc.) and, consequently, reduces the risk of intoxication and death. It should be noted that preserving the functional status of the intestine is one of the main tasks in coloproctology and oncology, since cancer patients, especially in severe stages with metastases, are usually accompanied by cachexia [17].

Separately, it should be emphasized that local fractional irradiation with local fractional electron irradiation at a summary dose of 25 Gy is available and controlled, has shown its efficacy, and can be used as a model for more detailed in vivo studies.

In conclusion, according to the results of morphological and immunohistochemical studies, the evaluation of the regenerative potential of the colonic epithelium and the status of progenitor cells in comparison with the literature’s data, electron irradiation is one of the most promising types, one of the advantages of which is a less-damaging effect on the tissues.

## 4. Methods and Materials

The animals—sexually mature Wistar rats (*n* = 60; body weight 200 ± 10 g)—were divided into two groups: I—control group (*n* = 10); II—experimental group (*n* = 20, local single electron irradiation at a dose of 2 Gy); III—experimental group (*n* = 30), with local fractional irradiation with electrons at local fractional electron irradiation at a summary dose of 25 Gy. The most commonly used doses were chosen (a single irradiation—2 Gy; a fractional irradiation—a summary dose of 25 Gy in five fractions of 5 Gy). The irradiation dose was selected according to the “Practical guidelines for drug treatment of colorectal cancer” (2020). Anaesthesia was administered before the procedure.

The rats were placed in a special “stretching” apparatus. The animals were irradiated in the Department of Radiation Biophysics of A.F. Tsyba MRSC (Obninsk, Russia) using a linear accelerator “NOVAC-11” (dose rate 1 Gy/min, energy 10 MeV, and frequency 9 Hz, field size Ø 40 mm).

Animals of the experimental group were removed from the experiment on the 5th day after the last irradiation.

After opening the abdominal cavity, the colon was carefully removed and fixed in buffered formalin, embedded in paraffin blocks, sectioned at 3 μm on a microtome and stained with hematoxylin and eosin. The number of crypts was assessed on the circumference of the colon.

Microscopic analyses were performed using a video microscopy system (Leica DM2000 microscope, Germany; Leica ICC50 HD camera).

Morphological changes were scored according to the following parameters:

(A) Degenerative changes: 0—absent; 1—mild; 2—moderate; 3—severe.
(B) Dystrophic changes of the epithelium: 0—absent; 1—hypertrophy of cells; 2—vacuolization of cytoplasm of up to 50% of cells; 3—degree of vacuolization of cytoplasm of all cells.
(C) Number of goblet cells: 0—absent; 1—mild reduction; 2—moderate reduction; 3—severe reduction.
(D) Number of smooth muscle cells: 0—absent; 1—degree of reduction/weak; 2—degree of reduction/moderate; 3—degree of reduction/severe.
(E) Inflammation: 0—absent; 1—mild (within the mucosa); 2—moderate (erythema, ulceration); 3—severe (deep ulceration).
(F) Macrophage infiltration: cell density per 1 mm^2^ was calculated.
(G) Vascularisation and full blood vessels: 0—absent; 1—congestion of up to three blood vessels; 2—congestion of three to five blood vessels; 3—congestion of more than five blood vessels.

The number of goblet cells, smooth muscle cells, and macrophages were counted per 1 mm^2^, and the results are reflected in the scores.

Morphometric analyses of haematoxylin and eosin-stained microspecimens were performed in 10 randomly selected fields of view of a light microscope at ×400 magnification, using a video microscopy system (Leica DM2000 microscope, Wetzlar, Germany) with Leica Application Suite, version 4.9.0. Computer morphometry was performed using an ImageJ computer-image-analysis system.

Each section of the colon, representing a segment, was carefully examined under the light microscope, taking into account the following histomorphometric parameters: organ length; wall thickness; diameter; depth of intestinal crypts; and diameter of the largest lymphoid follicle at maximum thickness. Diameters (external and internal) were calculated using the formulas D = C/π, A = rD2/4 wall thickness = serosal-mucosal diameter/2, taking into account the values of external (serosal) circumference and internal (mucosal) circumference. The above parameters were calculated in microns: mean luminal diameter, mean lymphoid follicle diameter, and mean serosal–mucosal circumference (wall thickness).

Immunohistochemistry was performed according to the standard protocol in the automatic mode of the Bond-Max immunohistostainer (Leica, Germany). Primary antibodies—against caspase-3 (Invitrogen/Thermo Fisher Scientific; 74T2, 1:50, Waltham, MA, USA) and Ki-67 (Abcam; ab15580, 1:100, Cambridge, UK); secondary antibodies—universal antibodies (HiDef Detection™ HRP polymer system, Cell Marque, Rocklin, California, USA). The number of immunopositive cells was counted in 10 fields of view at ×400 magnification. To estimate the proliferation index based on Ki-67, the percentage of stained cells per crypt was calculated.

The expression of the main members of the MAPK/ERK1,2 signalling pathway (ERK1, ERK2) and the PI3K/AKT signalling pathway (PIK3R1, AKT1, AKT2) was assessed using RT-PCR. We extracted cDNA for all the samples. Testis fragments were placed in a stabilizing solution and stored at −70 °C until testing. Samples were homogenized according to the standard protocol. Total RNA extraction was carried out using a set of ready-made reagents RNeasy Plus Mini Kit (QIAGEN, Venlo, The Netherlands). Complementary DNA (cDNA) synthesis was performed using Super Script™ VILO™ Master Mix (Invitrogen, Waltham, MA, USA). The isolated cDNAs were subjected to RT-PCR using a ready-made mixture of ABsolute Blue QPCR Mix reagents (ThermoFisher Scientific, Waltham, MA, USA) with SYBR Green I. RT-PCR was carried out using the Step One System (Applied Biosystems, ThermoFisher Scientific, Waltham, MA, USA) and standard software (StepOne™ Software v2.0.2). Gene expression analysis was performed using the threshold cycle (Ct) method and calculating relative gene expression according to the protocol. Control was performed against the housekeeping reference gene GAPDH. The selection of primers was carried out based on publicly available materials on DNA and mRNA sequences of genes in the NCBI database using Primer-BLAST (Table 4).

Western blot was used. Protein concentrations in all homogenized samples were determined using the Bradford method. A total of 25 μg of protein was then resolved on 12% SDS-PAGE and transferred to a nitrocellulose membrane (GeneTex, clone GTX110543, 1:600, Irvine, CA, USA). The membrane was blocked with 5% non-fat dry skim milk (Sigma Aldrich, 70166, Saint Louis, MO, USA) in Tris-buffered saline (TBS). Glyceraldehyde-3-phosphate dehydrogenase (GAPDH) immunostaining was used as a standard. The unit of measurement is nmol pNA/mg protein C. Visualization was carried out using a Novex ECL Reagents immunochemiluminescence kit (Invitrogen, Waltham, MA, USA).

The data obtained were processed using the SPSS 10.0 for Windows statistical-software package (IBM Analytics, Foster City, CA, USA), and the arithmetic mean, mean error, and Student’s criterion were calculated. Differences between means were considered significant at *p* ≤ 0.05.

## 5. Conclusions

Local single electron irradiation of the colon at a dose of 2 Gy resulted in a slight reduction in the number of goblet cells while maintaining their regenerative potential. On the other hand, local fractional electron irradiation of the colon with a total dose of 25 Gy caused mild degenerative–dystrophic changes in epithelial cells and a greater decrease in the number of goblet cells while retaining their regenerative potential. Injury to intestinal crypts results in a rise in caspase-3-positive epitheliocytes and a reduction in Ki-67 levels.

## Figures and Tables

**Table 1:** Morphological characteristics of the colon after local electron irradiation at doses 2 Gy and 25 Gy, in points.

	Control	2 Gy	25 Gy
Degenerative changes	0.00 ± 00	0.31 ± 0.10	2.37 ± 0.43
Dystrophic changes in the epithelium	0.00 ± 00	0.24 ± 0.06	1.30 ± 0.45
Number of goblet cells	0.00 ± 0.13	0.43 ± 0.21	1.64 ± 0.37
Number of muscle cells	0.00 ± 0.23	0.36 ± 0.35	1.58 ± 0.49
Number of macrophages	0.00 ± 0.21	0.41 ± 0.24	1.69± 0.29
Full-bloodedness	0.00 ± 00	0.27 ± 0.43	2.32 ± 0.51

**Table 2:** Morphometric characteristics of the colon after local electron irradiation at doses 2 Gy and 25 Gy.

Group	*n*	Organ Length, mm	Wall Thickness, µm	Outer Diameter, mm	Inner Diameter, µm
Control	10	10.7 ± 1.4	66.8 ± 25.9	3.1 ± 0.7	79.7 ± 20.4
2 Gy	20	10.1 ± 1.8	64.8 ± 24.6	3.0 ± 0.8	72.7 ± 20.9
25 Gy	30	9.3 ± 2.1	60.3 ± 22.6	2.8 ± 1.1	51.9 ± 21.7

**Table 3:** Number of IHC-positive colon epitheliocytes after local electron irradiation at doses 2 Gy and 25 Gy, %.

Marker	Control (I Group)	2 Gy (II Group)	25 Gy (III Group)
caspasa-3	14.5 ± 3.9	17.11 ± 4.79 ^a^	28.7 ± 8.2 ^b^
Ki-67	19.45 ± 5.12	16.34 ± 3.56 ^a^	11.14 ± 6.23 ^b^

^a^ control (I) и II group, ^b^ control (I) и III group; *p* < 0.05.

**Table 4:** RT-PCR primer sequence.

Gene	Direct Primer	Reverse Primer
*PIK3R1*	CCCTCAGTGGACTTGGATGT	GCTGCTGGGAATCTGAAAAG
*AKT1*	ACTCATTCCAGACCCACGAC	TGAGCTCGAACAGCTTCTCA
*AKT2*	ATGTAGACTCTCCAGATGAG	TGAGATAATCGAAGTCATTCA
*ERK1*	TCCCAAATCTGACTCCAAAGC	GCCACTGGTTCATCTGTCGG
*ERK2*	GGTTGTTCCCAAACGCTGAC	AATGGGCTCATCACTTGGGT
*GAPDH*	CCGTCTAGAAAAACCTGCC	AGCCAAATTCGTTGTCATACC

## Data Availability

The raw data supporting the conclusions of this article will be made available by the authors on request.
